# How Can Rotational Thromboelastometry as a Point-of-Care Method Be Useful for the Management of Secondary Thromboprophylaxis in High-Risk Pregnant Patients?

**DOI:** 10.3390/diagnostics11050828

**Published:** 2021-05-03

**Authors:** Lucia Stanciakova, Miroslava Dobrotova, Pavol Holly, Jana Zolkova, Lubica Vadelova, Ingrid Skornova, Jela Ivankova, Tomas Bolek, Matej Samos, Marian Grendar, Jan Danko, Peter Kubisz, Jan Stasko

**Affiliations:** 1 National Center of Hemostasis and Thrombosis, Department of Hematology and Transfusion Medicine, Jessenius Faculty of Medicine in Martin, Comenius University in Bratislava, Martin University Hospital, 03659 Martin, Slovakia; miroslava.dobrotova@gmail.com (M.D.); palhol@gmail.com (P.H.); jana.zolkova@gmail.com (J.Z.); lubka.vadelova@gmail.com (L.V.); inka.skornova@gmail.com (I.S.); ivankova@unm.sk (J.I.); kubisz@jfmed.uniba.sk (P.K.); stasko@jfmed.uniba.sk (J.S.); 2 Center of Immunology in Martin, 03601 Martin, Slovakia; 3 Department of Internal Medicine I., Jessenius Faculty of Medicine in Martin, Comenius University in Bratislava, Martin University Hospital, 03659 Martin, Slovakia; ato.bolek@gmail.com (T.B.); matej.samos@gmail.com (M.S.); 4 Biomedical center Martin, Laboratory of Bioinformatics and Biostatistics, Comenius University in Bratislava, Jessenius Faculty of Medicine in Martin, 03601 Martin, Slovakia; Marian.Grendar@uniba.sk; 5 Laboratory of Theoretical Methods, Institute of Measurement Science, Slovak Academy of Sciences, 84104 Karlova Ves, Slovakia; 6 Department of Gynecology and Obstetrics, Jessenius Faculty of Medicine in Martin and University Hospital in Martin, Comenius University in Bratislava, 03659 Martin, Slovakia; Jan.Danko@uniba.sk

**Keywords:** risky pregnancy, thromboembolism, hemostasis, rotational thromboelastometry

## Abstract

Thromboprophylaxis with low-molecular-weight heparin (LMWH) for patients with a history of venous thromboembolism (VTE) is suggested. Rotational thromboelastometry (ROTEM^®^) represents an innovative point-of-care method enabling the complex and quick evaluation of hemostasis. However, there are only episodic cases of its use for hemostasis assessment and guidance of LMWH in pregnancy. Therefore, we provide the results of unique prospective and longitudinal monitoring of hemostasis in high-risk pregnant women, which we used for the individualized optimalization of secondary thromboprophylaxis. According to the shortening of clot formation time (CFT) in EXTEM (*p* = 0.0007 from the 26th gestational week vs. controls) and INTEM (*p* = 0.002 from the 35th gestational week), increase in alpha angle (AA) in EXTEM, INTEM, and HEPTEM, and the persistence of increase in maximum clot firmness (MCF) in EXTEM, INTEM, and HEPTEM (*p* < 0.001 from the 26th and 35th gestational week vs. controls for EXTEM and INTEM, *p* = 0.0012 from the 26th gestational week in HEPTEM), LMWH dose was modified. Even after the postpartum period, AA in EXTEM was steeper than in controls (*p* = 0.0007), indicating that hemostasis is not fully normalized after 6–8 weeks following delivery. Therefore, ROTEM may be a useful tool for the individual evaluation of the termination of anticoagulant thromboprophylaxis.

## 1. Introduction

Physiological changes in pregnancy create a hypercoagulable state that is thought to be protective, especially at the time of delivery preventing excessive bleeding. Therefore, the presence of inherited or acquired thrombophilia increases the risk of development of venous thromboembolism (VTE) and adverse pregnancy outcomes due to vascular uteroplacental insufficiency [1].

VTE occurring in pregnancy is an important cause of maternal morbidity and mortality in developed countries. Its incidence represents 0.5–2.2 VTE events per 1000 pregnancies [2]. In comparison with the non-pregnant population of women of childbearing age, the relative risk is increased approximately fourfold. The risk in the course of the postpartum period is about fivefold higher than the risk during pregnancy. Previous superficial vein thrombosis is an independent risk factor for VTE during pregnancy or the postpartum period [3].

However, because of the low absolute frequency of episodes, we have only limited outcome-based clinical data. Therefore, the diagnosis and management of deep vein thrombosis (DVT), pulmonary embolism (PE), cerebral vein thrombosis, and dural sinus thrombosis are complicated by the clinical state of pregnancy and often require modifications of standard diagnostic and treatment algorithms in non-pregnant population [4].

According to the current recommendations of the American Society of Hematology (ASH) and the American College of Chest Physicians (ACCP), antepartum and postpartum thromboprophylaxis with low-molecular-weight heparin (LMWH) for all patients with a history of unprovoked or estrogen-associated VTE is suggested. For pregnant women with a provoked VTE episode and thrombophilia, individualized decision based on the type of provoked event and characteristics of thrombophilia should be made [5,6].

Rotational thromboelastometry (ROTEM^®^) represents a point-of-care method that enables the evaluation of viscoelastic profiles of whole blood, the processes of coagulation, and fibrinolysis [7,8]. ROTEM may detect prothrombotic changes associated with pregnancy. However, there is a limited evidence for its use in obstetric medicine. The hesitancy toward its more frequent use in this field of medicine may be because of the current deficiency of randomized controlled studies. Moreover, variability between study protocols and results highlights the need for further prospective studies to investigate its utility in the assessment of the risk for thrombosis and hemorrhage as well as in the management of thromboprophylaxis and treatment in pregnant women [8].

For all these reasons, here, we report the results of our unique prospective longitudinal study that evaluates the changes in ROTEM parameters in pregnant patients with a history of VTE using secondary anticoagulant thromboprophylaxis in order to prevent further VTE events. Based on the results, we could actually modify the dose of LMWH and increase its effectiveness, thus preventing thromboembolic events and pregnancy complications in high-risk pregnant patients and saving two lives on one occasion.

## 2. Materials and Methods

### 2.1. Patients

Forty-six pregnant women with a history of unprovoked or estrogen-associated thromboembolic event, with or without inherited thrombophilic state using LMWH as the secondary anticoagulant thromboprophylaxis, were included in the study.

The results of pregnant patients were compared with the control group that was composed of 54 healthy women without personal or family history of thromboembolism, pregnancy complications, such as placental abruption, repeated pregnancy loss, intrauterine growth restriction (IUGR), fetal dismiss, or VTE in the course of pregnancy. These subjects did not take any drugs that might influence hemostasis—anticoagulants, antiplatelet agents, oral contraceptives, etc.

### 2.2. Study Design

Prior the clinical examination, an atraumatic blood sampling of fasting pregnant patient into Vacutainer^®^ blood collection tube with anticoagulation reagent (3.2% sodium citrate) for the analysis performed by rotational thromboelastometer ROTEM^®^ delta (Pentapharm GmbH, Munich, Germany) was done. Due to the standardization of the monitoring of the “peak” LMWH activity, the pregnant patients were asked to administer LMWH 3 h before taking of blood.

Blood samples were taken during 5 time intervals: T1 at the 10th–12th week of pregnancy, T2 at the 16th–18th week of pregnancy, T3 at the 26th–28th week of pregnancy, T4 at the 35th–36th week of pregnancy, and T5 during the 6th–8th week after the delivery. At the T1 control, the patient reported the data about personal and family history, allergy, drugs, and gynecological history (previous deliveries, their form (spontaneous or cesarean section, complications, abortions, interruptions, etc.).

After processing of the obtained data, values of clotting time (CT), clot formation time (CFT), maximum clot firmness (MCF), and alpha angle (AA) described in detail below were compared between some time interval during pregnancy (T1–T4), and the results were obtained after the postpartum period (T5) when the levels of measured parameters are presumed to be normalized. The results of the patients were also compared with the data of the control group.

### 2.3. Rotational Thromboelastometry

Thromboelastography was firstly described in 1948 by H. Hartert as a method able to evaluate the viscoelastic features of coagulation and subsequent fibrinolysis in whole blood sample under low shear conditions (approximately 0.1/s) similar to these present in areas of the venous part of circulation, venules, large veins, such as vena cava and in the arterial system [9,10,11,12,13].

**Figure 1:**
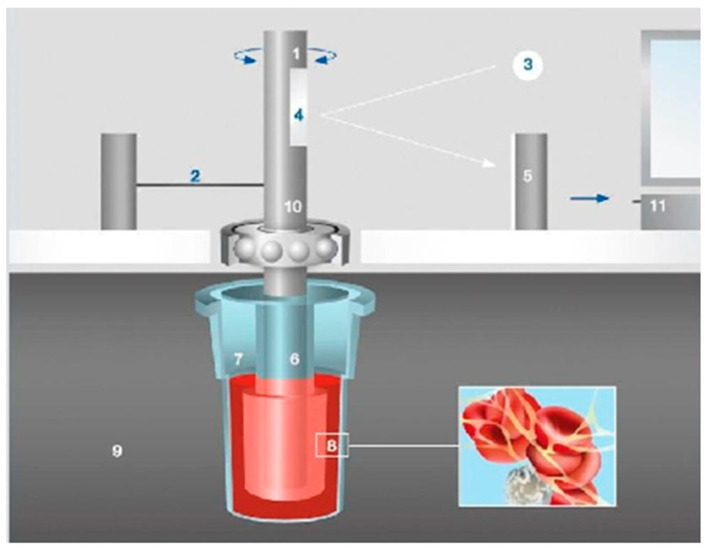
The principle of ROTEM analysis [10]. Legend: 1—oscillating axis, 2—counterforce Scheme, 3—light beam from LED, 4—mirror, 5—detector (electronic camera), 6—sensor pin, 7—cuvette filled with blood sample, 8—fibrin strands and platelet aggregates, 9—heated cuvette holder, 10—ball bearing, 11—data processing unit (adapted from [10]).

For the ROTEM analysis in our study, 300 μL of citrated whole blood was used [14]. Blood was incubated at 37 °C and placed in the cup [9]. The ROTEM machine required approximately 4 min for warming up [14]. At the beginning of each measurement, a pin connected to an optical detector was suspended within the cup [9]. Initially, a cylindrical cup remained fixed, and a pin suspended on a ball bearing mechanism oscillated in the angle 4°75′ every 6 s (s) applying a constant force [10]. However, as fibrin was formed between them, the impedance of the rotation of the pin was detected, and a trace was generated, as illustrated in Figure 1 [9,10].

**Figure 2:**
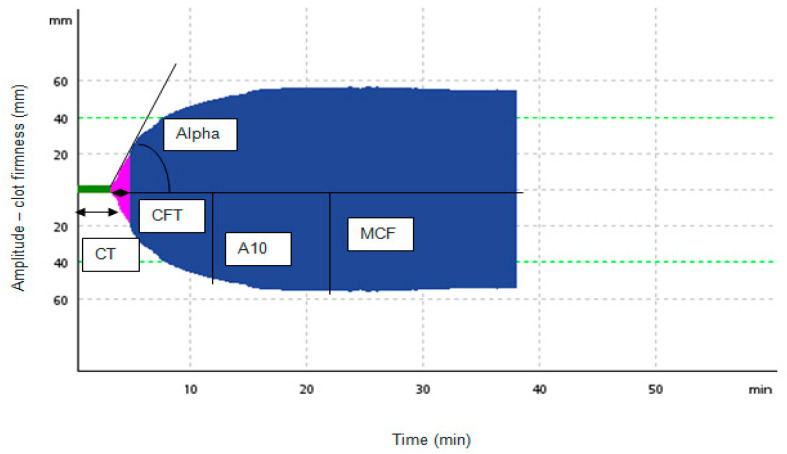
Selected ROTEM parameters. Legend: A10–amplitude at 10 min after CT, alpha—alpha angle (AA), CFT—clot formation time, CT—clotting time, MCF—maximum clot firmness (modified with the use of the authors’ data)**.**

The trace was composed of several parts that reflect various stages of the hemostatic process [9]. General definitions and characteristics of the measured parameters and their reference ranges for particular tests used in the study are compiled in Table 1. Additionally, A5 (in mm) is the amplitude at time point 5 min after CT. Analogically, A10 (in mm) is the amplitude achieved 10 min after CT [14]. Lysis Index 30 (LI30) is defined as the percentual reduction in MCF that develops when amplitude is measured 30 min after CT is detected [10]. The example of the curve with the outline of the described parameters is illustrated in Figure 2.

We used various ROTEM^®^ tests for different purposes. At the beginning, star-tem reagent containing the optimized amount of calcium chloride for recalcifying of citrated blood was added to the blood sample. Then, recalcified blood was analyzed using the activated tests EXTEM, INTEM, and HEPTEM (star-TEM^®^, Pentapharm GmbH) (Table 1) [14].

According to the information of the manufacturer, reference ranges for parameters obtained by the HEPTEM test are not determined. There is only a statement indicating that comparison of HEPTEM and INTEM enables the differentiation of the effect of heparin on coagulation from other factors, such as dilution, coagulation factor deficiency, fibrinogen content, or platelet count and also that after removal of heparin using the hep-TEM^®^ reagent, coagulation parameters obtained with ROTEM^®^ can be used to assess hemostasis. However, according the study of Lang et al. and Tanaka et al., the reference ranges for parameters obtained by INTEM can be used also for HEPTEM (see Table 1) [19,20]. Therefore, we added their reference ranges for HEPTEM test derived from those valid for INTEM test in the Table 1. Anyway, the reference values for AA are not available.

### 2.4. Statistical Analysis

The study consisted of a longitudinal component (measurements of the patient cohort at the five time points) and a cross-sectional component, where the same patient cohort was compared to the control group. The data from the cross-sectional part of the study were visualized by a swarmplot overlaid on a boxplot. The data from the longitudinal component of the study were visualized by the spaghetti plot. The data were summarized by median and the lower, upper quartiles, due to the presence of outliers. Prior to all formal statistical analyses, outliers were identified by the Hampel filter (median +/− 3 * median absolute deviation) and removed from further considerations.

The ANOVA test was applied to test the null hypothesis of the equality of the population means of a variable for the five time points and the control group, which was followed by the post hoc pair comparisons of the five time points and the control group. The five post hoc hypotheses were specified as the linear restrictions (T1 − Co = 0, T5 − Co = 0). Simultaneously, adjusted post hoc 95% confidence intervals were used to quantify the uncertainty of the difference of the population means. The *p*-values of post hoc tests were adjusted by the single-step method.

Data from the longitudinal component of the study were modeled by the linear mixed model with patients as the random effect. Type III ANOVA test with Satterthwaite’s method was followed by computations of the post hoc confidence intervals with adjustment by the Sidak method; *p*-values for the pairwise comparisons were adjusted by the Tukey method.

The data from INTEM and HEPTEM longitudinal studies were used in a linear mixed model with time, type (INTEM or HEPTEM), and their interaction as the fixed effects and patients as the random effect. A type III ANOVA test was used to test the significance of predictors. In the case of significant effect, the test was followed by post hoc pairwise comparisons of INTEM and HEPTEM at the five time points.

All the linear mixed models were subjected to standard diagnostics of residuals. Results with the adjusted *p*-value below 0.05 were considered statistically significant. The data were analyzed in R [21], ver. 4.0.4 (R Foundation for Statistical Computing, Vienna, Austria), using libraries multcomp [22], lme4 [23], emmeans [24], and CGP functions [25].

### 2.5. Ethical Approval

The study was conducted according to the guidelines of the Declaration of Helsinki and approved by the Ethics Committee of Jessenius Faculty of Medicine in Martin, Comenius University in Bratislava (approved on 11 December 2013 with the protocol code EK 1422/2013).

## 3. Results

Baseline characteristics of the pregnant patient group and the control group are outlined in Table 2. Half (50%) of the patients had positive family history. Inherited trombophilic mutations, such as Factor V Leiden and prothrombin variant G20210A were detected in 11.72% and 0.9% of all pregnant women. The most frequent acquired thrombophilic state was protein S (PS) deficiency present in 26.13% of the patients. The second most common acquired thrombophilia was increased FVIII activity developed in 18.92%, and the third most prominent prothrombotic change was activated protein C resistance (APCR), occurring in 15.32% of included individuals.

The development of prothrombotic changes in hemostasis that increases the risk of the repeated thromboembolic episode was detected in the majority of studied parameters.

### 3.1. EXTEM

All calculated median values remained in the reference range for CT in EXTEM during the whole studied period of time.

When comparing the results between T5 and values detected in T1–T4, no statistically significant results were obtained. Similarly, the comparison between control group and T1–T5 was not statistically significant (Table 3). Moreover, according to the data obtained by the longitudinal component of the study, there was not any statistically significant difference in the comparison between described five time points of blood sampling.

**Figure 3:**
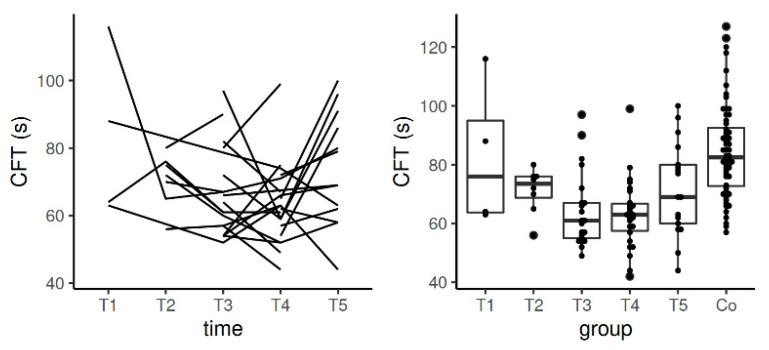
Spaghetti plot of the data from the longitudinal study (**left**), and swarmplot with boxplot of the same data together with the data from the control group (**right**)—the results of CFT in EXTEM. Legend: CFT—clot formation time, Co—control group, T1—time interval 1, T2—time interval 2, T3—time interval 3, T4—time interval 4, T5—time interval 5.

Median values of CFT in EXTEM during pregnancy and after the postpartum period stayed also in the reference range determined by the manufacturer according to the values obtained in the non-pregnant population. Significant difference between the population means of CFT for T3 and control group (*p* = 0.0007) and T4 and controls (*p* = 0.0027) was observed. Data from the longitudinal component of the study (measurements of the patient cohort at the five time points; see Figure 3) showed statistically significant difference of the population means of CFT in the comparison between T1 and T3 (*p* = 0.02) and between T1 and T4 (*p* = 0.01).

**Figure 4:**
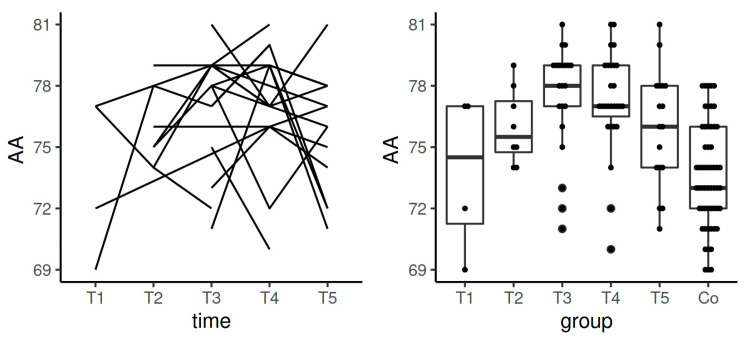
Spaghetti plot of the data from the longitudinal study (**left**), and swarmplot with boxplot of the same data together with the data from the control group (**right**)—the results of AA in EXTEM. Legend: AA—alpha angle, Co—control group, T1—time interval 1, T2—time interval 2, T3—time interval 3, T4—time interval 4, T5—time interval 5.

The values of AA in EXTEM stayed in the reference range in the course of the whole study including pregnancy and the postpartum period. Significant differences were detected between the control group and T3–T5 (*p*-value in the comparisons between the control group and T3 and controls with T4 was <0.0001; when comparing controls and T5, *p*-value was 0.0007). Data from the longitudinal component of the study confirmed statistically significant difference in the comparison between T1 and T3 (*p* = 0.025) (Table 3, Figure 4).

**Figure 5:**
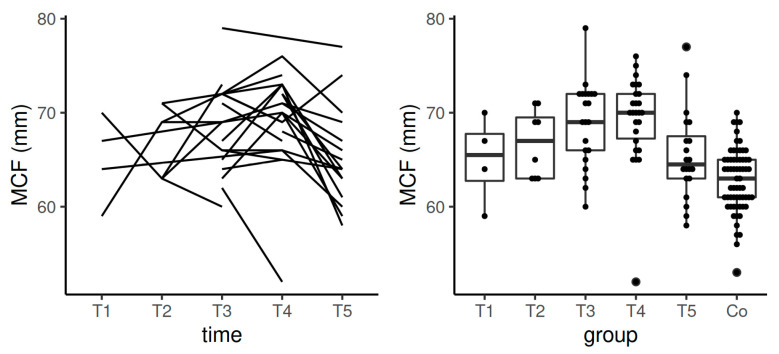
Spaghetti plot of the data from the longitudinal study (**left**), and swarmplot with boxplot of the same data together with the data from the control group (**right**)—the results of MCF in EXTEM. Legend: Co—control group, MCF—maximum clot firmness, T1—time interval 1, T2—time interval 2, T3—time interval 3, T4—time interval 4, T5—time interval 5.

There was a significant difference of MCF values in EXTEM between T3 and T4 vs. control group (*p* < 0.001 for both comparisons). MCF values stayed in the reference range determined by the manufacturer according to the values obtained in the non-pregnant population. According to the data obtained by the longitudinal component of the study, a statistically significant difference in the comparison between T3 and T5 (*p* = 0.01) and between T4 and T5 (*p* = 0.0005) was observed (Table 3, Figure 5).

### 3.2. INTEM

In INTEM, CT stayed in the reference range determined by the manufacturer according to the values obtained in the non-pregnant population. We did not detect any statistically significant results in the comparison between the high-risk pregnant patients and the control group. Data from the longitudinal component of the study showed statistically significant difference in the comparison between T1 and T3 (*p* = 0.016), between T1 and T4 (*p =* 0.01), and after the comparison of T4 and T5 (*p* = 0.003) (Table 4).

**Figure 6:**
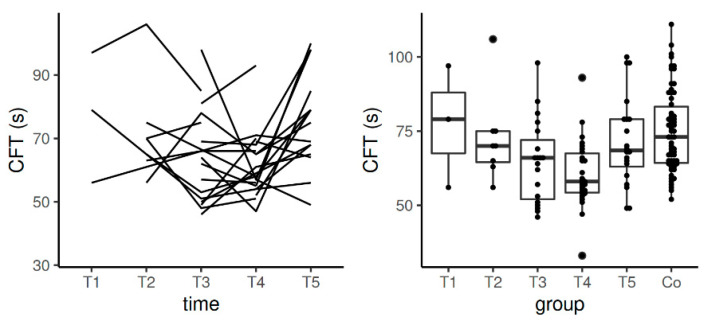
Spaghetti plot of the data from the longitudinal study (**left**), and swarmplot with boxplot of the same data together with the data from the control group (**right**)—the results of CFT in INTEM. Legend: CFT—clot formation time, Co—control group, T1—time interval 1, T2—time interval 2, T3—time interval 3, T4—time interval 4, T5—time interval 5.

CFT values in INTEM stayed in the reference range determined by the manufacturer according to the values obtained in the non-pregnant population. Statistically significant data were obtained in the comparison between the control group and T1 (*p* = 0.0024) and between the controls and T4 (*p* = 0.002). Data from the longitudinal component of the study confirmed statistically significant difference in the comparison between T4 and T5 (*p* = 0.006) (Table 4, Figure 6).

**Figure 7:**
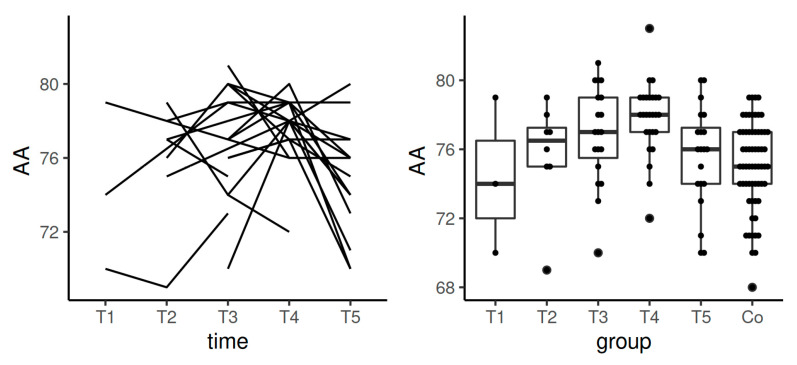
Spaghetti plot of the data from the longitudinal study (**left**), and swarmplot with boxplot of the same data together with the data from the control group (**right**)—the results of AA in INTEM. Legend: AA—alpha angle, Co—control group, T1—time interval 1, T2—time interval 2, T3—time interval 3, T4—time interval 4, T5—time interval 5.

Median values for AA in INTEM stayed in the reference range during the whole studied period of the time. We observed significant differences between the control group and T4 (*p* = 0.0019). Data from the longitudinal component of the study confirmed statistically significant difference in the comparison between T4 and T5 (*p* *=* 0.006) (Table 4, Figure 7).

**Figure 8:**
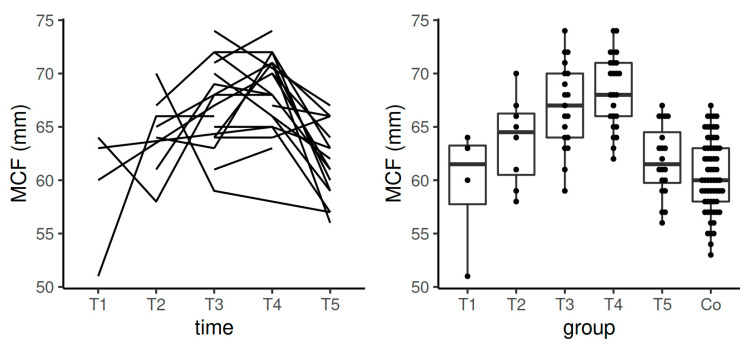
Spaghetti plot of the data from the longitudinal study (**left**), and swarmplot with boxplot of the same data together with the data from the control group (**right**)—the results of MCF in INTEM. Legend: Co—control group, MCF—maximum clot firmness, T1—time interval 1, T2—time interval 2, T3—time interval 3, T4—time interval 4, T5—time interval 5.

Median values for MCF in INTEM in the course of pregnancy, after the postpartum period and in the control group stayed also in the reference range (Table 4, Figure 8). To be more exact, significant results were obtained in the comparisons between the control group and T3 and between controls and T4 (*p*-values for both comparisons < 0.0001). According to the data obtained by the longitudinal component of the study, there was a statistically significant difference in the comparison between T1 and T3 (*p* = 0.0004), T1 and T4 (*p* = 0.0005), T2 and T4 (*p* = 0.018), T3 and T5 (*p* = 0.0005), and between T4 and T5 (*p* = 0.000) (Table 4, Figure 8).

### 3.3. HEPTEM

No statistically significant difference of CT in HEPTEM in the comparison between particular time intervals of blood sampling during pregnancy and postpartum period and the control group was achieved. Moreover, according to the data obtained by the longitudinal component of the study, there was not any statistically significant difference in the comparison between values of CT, CFT, and AA in described five time points of blood sampling (Table 5).

Medians of the CFT values in HEPTEM obtained in our study after the postpartum period were comparable with the median of CFT in the control group (Table 4). However, we did not calculate significant values.

**Figure 9:**
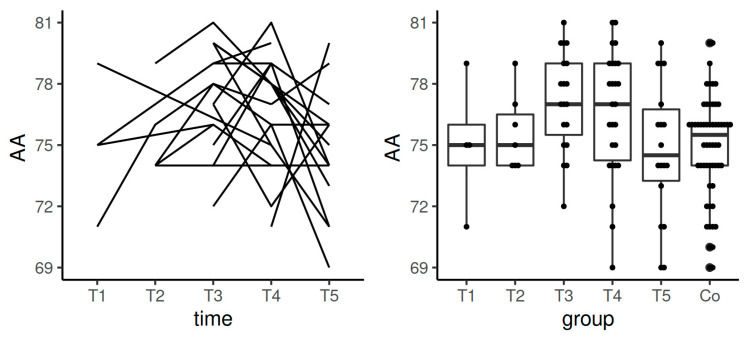
Spaghetti plot of the data from the longitudinal study (**left**) and swarmplot with boxplot of the same data together with the data from the control group (**right**)—the results of AA in HEPTEM. Legend: AA—alpha angle, Co—control group, T1—time interval 1, T2—time interval 2, T3—time interval 3, T4—time interval 4, T5—time interval 5.

Median values of AA in HEPTEM increased during pregnancy, achieving statistically significant results in the comparison between the control group and T3 (*p* = 0.02) (Table 5, Figure 9).

**Figure 10:**
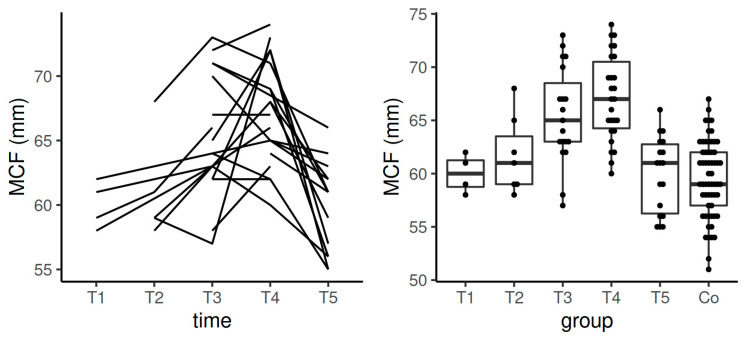
Spaghetti plot of the data from the longitudinal study (**left**) and swarmplot with boxplot of the same data together with the data from the control group (**right**)—the results of MCF in HEPTEM. Legend: Co—control group, MCF—maximum clot firmness, T1—time interval 1, T2—time interval 2, T3—time interval 3, T4—time interval 4, T5—time interval 5.

We obtained statistically significant results of MCF in HEPTEM in the comparison between the control group and T3 (*p* = 0.0012) and controls and T4 (*p* < 0.0001). Moreover, there was a significant difference between T2 and T4 (*p* = 0.0282). Data from the longitudinal component of the study confirmed a statistically significant difference in the comparisons between T1 and T4 (*p* = 0.003), T2 and T4 (*p* = 0.004), T3 and T5, and T4 vs. T5 (*p*-value for both comparisons is 0.000) (Table 5, Figure 10).

### 3.4. Is the Time Evolution of CT, CFT, AA, and MCF Different in INTEM Than in HEPTEM?

The data from INTEM and HEPTEM longitudinal studies were used in a linear mixed model to model the association between a measured parameter (CT, CFT, AA, MFT) and time (T1, T2, T3, T4, T5), type (INTEM or HEPTEM) and their interaction.

For CT, CFT, and AA, only the time interaction was proved to be statistically significant (*p* = 0.00038, <0.0001 and 0.0002, respectively). Neither type of the test nor the time and type interactions were significant. In CT, type of the test (INTEM or HEPTEM) was not significant (*p* = 0.11) nor was the interaction (*p* = 0.46). Regarding CFT, type of the test was not significant (*p* = 0.34) nor was the interaction (*p* = 0.77). In AA, again, the type of the test was not significant (*p* = 0.47) nor was the interaction (*p* = 0.62).

**Figure 11:**
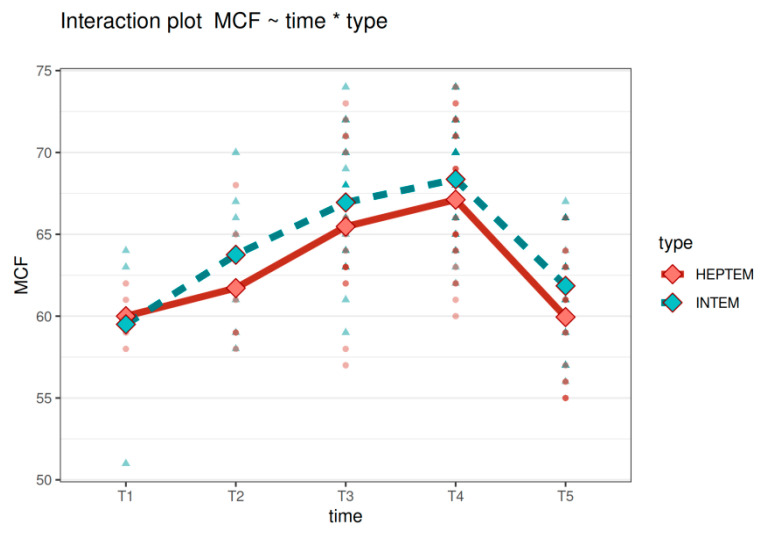
The interaction plot for MCF in HEPTEM and INTEM. In addition to the data points, the plot depicts the average value of MCF for HEPTEM (in red) and INTEM (in green) at different time points. Legend: MCF—maximum clot firmness, T1—time interval 1, T2—time interval 2, T3—time interval 3, T4—time interval 4, T5—time interval 5.

On the contrary with these results, in MCF (see Figure 11), besides the time interaction (*p* < 0.0001), the type of the test was also statistically significant (*p* = 0.048)—a post hoc analysis showed the mean MCF in T5 in HEPTEM to be significantly lower than in INTEM (*p* = 0.023; decrease by −2.0 in the average). However, the interaction between time and type was not statistically significant (*p* = 0.84).

### 3.5. Clinical Data

Healthy newborns were delivered in all patients; mean gestational week at the time of delivery was the 24th week. Despite anticoagulant thromboprophylaxis modified according to the obtained results, we observed the recurrence of VTE in the 15th week of pregnancy in one patient. This pregnant woman was subsequently excluded from the study, because she was managed with the therapeutic dose of LMWH. The administration of LMWH in the included patients was well tolerated without serious side effects. Allergic reaction manifested as a rash caused the change of the product of LMWH with its different basic substance (enoxaparin, nadroparin, or dalteparin) and was found in 47.83%. We did not notice any renal impairment in the studied patients. Average levels of liver enzymes were in the reference range, and none of the patients developed HELLP syndrome and heparin-induced thrombocytopenia. Moreover, fibrinogen levels also stayed in the reference range for a whole period of the study (Table 2).

## 4. Discussion

The most important benefit of ROTEM method is the quick availability of the results, decreased sensitivity to mechanical stress and vibration, as well as improved reproducibility. The data are continuous, digital, and available for further calculations [26]. Moreover, ROTEM as the point-of-care method has the capacity to measure the whole coagulation process, initiated by fibrin formation and continuing through to clot retraction and fibrinolysis at the patient’s bedside with minimal delay. Last but not least, the coagulation status of the patient is evaluated in whole blood, enabling the interaction of the plasmatic coagulation system with red blood cells and platelets, thus providing useful information about platelet function. With all of these advantages, ROTEM provides helpful information in a wide scale of clinical situations, e.g., massive hemorrhage, evaluation of hypo- and hypercoagulable states, guiding pro- and anticoagulant therapy, in cardiac and liver surgery, or in diagnostics of a surgical bleeding [27].

In pregnancy, ROTEM has been used for the transfusion and coagulation management in the life-threatening cases of postpartum hemorrhage [28,29,30,31,32,33,34,35,36], in the presence of congenital or acquired bleeding disorders [37,38,39,40] including the detection of hyperfibrinolysis and amniotic fluid embolism [41,42,43,44,45].

In several cases, even though the variations of ROTEM values were within the reference range, they had the clinical meaning in terms of an increased risk of bleeding—for instance, women either with or without postpartum hemorrhage had similar median values of MCF in the FIBTEM test (23 mm vs. 23 mm, *p* = 0.710) [31]. According to the study of Yoon, the use of hydroxyethyl starch in women undergoing cesarean section can prolong the clot reaction time and kinetic time in thromboelastography, although their values were still within the reference range [38]. Similarly, in our study, despite the use of LMWH in high-risk pregnant patients, results of ROTEM analysis were in the reference range.

On the other hand, ROTEM may detect the hypercoagulable state associated with pregnancy [8]. Lee et al. performed a prospective observational study of baseline parameters for ROTEM in healthy laboring women with uncomplicated pregnancy concluding that FIBTEM, EXTEM, and INTEM amplitudes were higher at term gestation than the determined reference ranges for the non-obstetric population [46]. The same authors evaluated baseline parameters of ROTEM in healthy pregnant women undergoing elective caesarean section. When compared to the manufacturer’s reference ranges for the non-pregnant population, similarly as in the previous study, the FIBTEM MCF and FIBTEM, EXTEM, and INTEM amplitudes were higher. The results of this study showed an increase in coagulability in the course of normal pregnancy in the comparison with reference ranges obtained from the non-pregnant patients [47]. Published reference values for ROTEM parameters after non-hemorrhagic deliveries were correlated with the standard parameters of hemostasis [48,49].

ROTEM^®^ was used also for the assessment of the concomitant effect of pregnancy and obesity on hemostasis in healthy pregnant patients presenting for elective caesarean section at term. However, no association between BMI and ROTEM^®^ parameters in studied pregnant women was found [50].

In another study, a significant decrease in the mean time-to-clotting parameters in laboring patients compared with non-laboring patients was confirmed. Mean values for markers of clot firmness were higher in laboring patients, confirming increased hypercoagulability in laboring patients [51].

All studies described above were performed only at the time of delivery (peripartum period) and/or compared with non-pregnant population. Even though the reference ranges for ROTEM^®^ parameters in the patients during delivery were published [49], they are not relevant for the comparison with our results due to the peripartal bleeding and related activation of hemostasis. Moreover, such values were obtained at the exact time point of delivery; thus, they are incomparable with our closest time points T4 and T5 that were designed in the 35th–36th week of pregnancy and 6–8 weeks after delivery.

According to Amgalan et al., to date, there are only two randomized controlled trials on the use of thromboelastography (TEG)/ROTEM in obstetrics [8]. The results of such prospective longitudinal studies of the patients with uncomplicated pregnancies and after their postpartum period confirmed that with increasing gestational age, there are significant changes toward hypercoagulable state [52,53].

However, there is only a limited amount of studies using ROTEM for the evaluation of prothrombotic changes of hemostasis in pregnancy of patients with a history of a thromboembolic event and for the management of their secondary anticoagulant thromboprophylaxis. In the available literature, we found only episodic cases of the use of ROTEM for the hemostasis assessment and subsequent guidance of the use of LMWH in such high-risk pregnancy. In the case report of pregnant woman with antiphospholipid syndrome and HELLP syndrome who underwent a cesarean section 9 h after the use of heparin, HEPTEM assay helped to confirm heparin neutralization and manage intraoperative transfusion administration [54]. In another study of 97 women undergoing cesarean delivery in the presence or absence of LMWH, coagulation tests including ROTEM revealed hypercoagulation present after delivery and a tendency toward the normalization of hemostasis during the early postpartum period. The results in the group of patients receiving thromboprophylaxis showed a higher amount of coagulation parameters within the reference range [55].

In our study, we followed up pregnant patients longitudinally and prospectively, comparing the results obtained at particular time points during pregnancy with the results measured after the postpartum period, when it is presumed that they will be normalized. Simultaneously, we compared the results with the control group of non-pregnant patients, as did the studies described above. Generally, we observed the development of prothrombotic changes in hemostasis in the course of pregnancy with only partial normalization after the postpartum period.

In EXTEM, CT stayed in the reference range determined in the non-pregnant population, and we did not obtain significant results in the comparisons between the blood samplings during pregnancy (T1–T4) and after the postpartum period (T5). Moreover, there were no significant differences between the studied population and the control group. However, when looking at the calculated median values, prolongation of CT in T2 was potentially caused by the onset of the effect of LMWH after its repetitive administration in the course of pregnancy. Similarly, the prolongation of CT in T4 was probably due to the onset of the effect of the increased dose of LMWH after its common adjustment in T3. On the other hand, the shortening of CT in T3 was probably caused by the accompanying development of acquired changes of hemostasis and along with them led to the increase in LMWH dose (as mentioned in the Results section of this article, the increase in the dose of LMWH was recommended most frequently in T3 in 35.59% of the pregnant patients) (Table 2).

For CFT in EXTEM, median values in T5 were shorter than in non-pregnant control group and shorter than in T1 (Table 3, Figure 3). This can indicate that we should evaluate clinical factors and laboratory results obtained in these patients in a complex way and assess the termination of anticoagulant thromboprophylaxis individually, because hemostasis may not be fully normalized after the postpartum period.

AA is steeper in pregnancy, and thus, the amplitude of clot firmness is thicker and the formed clots were firmer and stronger than in the control group. The dynamics of this parameter (Table 3, Figure 4) also point out that hemostasis is not fully normalized even in T5 (after the postpartum period).

In EXTEM, MCF increased during pregnancy with the decrease in T5 to values comparable with T1 but still not achieving the values in the control group (Table 3, Figure 5). In the correlation with the above-analyzed results, this highlights the incompleteness of the normalization of hemostasis after the postpartum period.

Based on the shortening of CT in INTEM during pregnancy (Table 4), the increase in the LMWH dose was recommended mostly in T3.

Median value of CFT in INTEM in T5 (after the postpartum period) achieved the results from the control group. However, the most significant shortening of this time was observed in T4. This may indicate the need to evaluate the increase of the dose of thromboprophylaxis before the delivery (Table 4, Figure 6). As comparable with EXTEM, a similar tendency (the increase of AA values during pregnancy in INTEM and HEPTEM) points to the increasing prothrombotic tendency with advancing gestational age (Table 4 and Table 5, Figure 7 and Figure 9).

Significant comparisons between the results of MCF in INTEM the control group and T3 and T4 in the cross-sectional part of the study and majority of the comparisons in its longitudinal part with T4 and T5 confirm the progressive thrombophilic tendency developed in high-risk pregnant women despite the use of the thromboprophylaxis. Moreover, again, incomplete normalization of hemostasis can warn the clinicians before the preterm withdrawal of LMWH and justify individualized management of risky pregnant patients.

Again, a decrease of CT in HEPTEM in T3 may be the indicator of more prominent activation of hemostasis present despite the use of anticoagulant thromboprophylaxis and can represent one of the reasons to increase the dose of LMWH during this phase of pregnancy (26th–28th week) (Table 5).

Significant results of the comparisons of MCF parameter in HEPTEM test between the controls and T3 and T4, as well as the majority of the comparisons in the longitudinal part of the study with T4 and T5 indicate the increasing thickness of the blood of high-risk pregnant patients with the risk of the recurrence of the thromboembolic episode (Table 5, Figure 10).

To evaluate the effect of heparin, it is recommended to use HEPTEM in conjunction with INTEM [10]. Thus, also in our study, in the association with time, comparing the results of INTEM and HEPTEM, we obtained significant differences in all analyzed parameters. MCF in this analysis seems to be the most reliable parameter, because it was significantly higher in INTEM than in HEPTEM (*p* = 0.023) and confirmed the significance also with regard to the type of the test.

To generalize these results, statistically significant differences were the most common between the control group and T4 (or T3, respectively), when hemostasis becomes more activated despite the use of LMWH—this could be observed in the dynamics of CFT, AA, and MCF in EXTEM, in CT, CFT, AA, and MCF in INTEM, and in AA and MCF in HEPTEM. Therefore, the most frequent adjustment of the LMWH dose was recommended in T3 and T4.

After the calculation of the median values in our study and their comparison with reference values for particular parameters obtained in non-pregnant population, we can state that all our results stayed in the reference range. On the contrary to the conclusions of the study of Lee et al. [46,47], who confirmed higher amplitudes in studied individuals when compared with the non-pregnant population, in our study, the results stayed normal probably due to the use of the anticoagulant thromboprophylaxis. However, the reference ranges were determined in the non-pregnant population, so they could be evaluated in a different way using the ranges for the (high-risk) pregnant population.

When looking at the dynamics of the results obtained in our study, we can conclude that according to the persistence of the shortening of CT in EXTEM and INTEM, CFT in EXTEM, INTEM, and HEPTEM, the presence of the increase in AA in EXTEM, INTEM, and HEPTEM and the persistence of the increase in MCF in EXTEM, INTEM, and HEPTEM in T5, hemostasis is not normalized even after the postpartum period of 6–8 weeks after the delivery. Even when excluding the influence of heparin by the performance of HEPTEM in the comparison to INTEM [56], prothrombotic changes of hemostasis in EXTEM and significant results of MCF in HEPTEM are still present. This extends the period of time after delivery when the hemostasis in not normalized, as initially confirmed after three weeks following delivery in the study of Saha et al. [57].

The initial dose of LMWH that was used in our high-risk pregnant patients was 0.3–0.6 mL administered once daily and depending on the initial weight of the pregnant patient. Change of the dose of LMWH in a particular patient included in our study was recommended according to the increasing weight, after the observation of the significant change of the majority of ROTEM parameters in the comparison with patient’s results from the previous time point of blood sampling, according to the comparison with median results of ROTEM parameters of the same time point of blood sampling after the enrollment of a sufficient number of patients, according to the observation of the development of significant acquired changes in hemostasis (e.g., significant increase in FVIII activity, decrease of function of PS, antithrombin, development of APCR) or in the correlation with anti-Xa activity for LMWH. From one to another time point of blood sampling, an increase of only 0.1–0.2 mL of LMWH was recommended.

According to the frequency of significant changes, the most important parameters of ROTEM seem to be CFT, AA, and MCF, but not CT. However, we were not strictly focused only on the significant changes in one particular parameter of ROTEM analysis.

For the decision on the withdrawal of LMWH, we used the comparison of the patient´s data with the results of the control group and after the enrolment of sufficient number of the patients in T5 also the results of T5. Due to the persistence of the prothrombotic changes of hemostasis described above, such withdrawal of LMWH was postponed even after the postpartum period in 21.74% of the studied patients.

If our patients were not treated with LMWH or if the dose was not sufficient, they would be endangered by the recurrence of the VTE event. All of them experienced such episodes in the past, and currently, pregnancy increases their prothrombotic risk again. As it can be seen on the example of one patient described in the Results section, despite anticoagulant thromboprophylaxis modified according to the obtained results, the recurrence of VTE in the 15th week of pregnancy was diagnosed. As the thrombosis may be developed also in the uteroplacental circulation, one of the possible consequences of the inadequate dosage of LMWH might be intrauterine fetal death or IUGR caused by total or partial obstruction of this part of the circulation by the thrombosis. In the laboratory markers, without the adjustment of the dose of LMWH, the activation of hemostasis could be developed with potential shortening of the standard coagulation tests; an increase of D-dimers or FVIII, decrease of PS activity, and lower anti-Xa activity could be detected.

Therefore, along with the assessment of the further hemostatic parameters (standard and advanced coagulation tests, such as the control of the development of acquired prothrombotic changes of hemostasis—increase in FVIII activity, decrease of PS function, APCR, etc. or in the correlation with anti-Xa activity) and clinical state of the patient, the hematologists taking care of high-risk pregnant patients with the history of thromboembolic event should very carefully and individually evaluate the exact time period of termination of secondary anticoagulant thromboprophylaxis. Our results pose the question of when it is the right time for the withdrawal of such thromboprophylaxis and indicate that this time is strictly individual and does not have to be usual after the postpartum period.

## 5. Conclusions

In the manufacturer’s information about ROTEM reagents, there are reference ranges calculated in the healthy non-pregnant population. Moreover, in the available sources of literature on the use of ROTEM in obstetrics, mostly the reports about the use of ROTEM in the management of postpartum hemorrhage have been published. Due to the possibility of the development of this life-threatening condition, some of the authors processed the results of the healthy pregnant patients particularly at the time of delivery, thus providing the “reference values” for the hemorrhagic scenarios.

However, only episodic data have been published about the use of ROTEM in the pregnant patients with a risk of the occurrence or the recurrence of the thromboembolic event. Therefore, we present a study of the high-risk pregnant patients providing longitudinal assessment of their ROTEM results. Based on the comparison with healthy non-pregnant patients, despite the use of LMWH and stability of the results in the published reference ranges determined for the non-pregnant population, the normalization of hemostasis after the postpartum period is not fully achieved.

The most important results of the study are statistically significant shortening of CFT in EXTEM (*p* = 0.0007 from the 26th gestational week (time point T3) vs. controls) and INTEM (*p* = 0.002 from the 35th gestational week (time point T4)), increase in AA in EXTEM, INTEM, and HEPTEM and the persistence of increase in MCF in EXTEM, INTEM, and HEPTEM (*p* < 0.001 from the 26th and 35th gestational week vs. controls for EXTEM and INTEM, *p* = 0.0012 from the 26th gestational week in HEPTEM).

Moreover, according to the data obtained by the longitudinal component of the study, we obtained statistically significant differences mainly in the comparisons between T1 vs. T3 and T1 vs. T4 or T3 vs. T5 and T4 vs. T5, showing the differences between the beginning of the pregnancy represented by T1 (or between the time point T5 after the postpartum period, when the normalization of hemostasis can be awaited) vs. advanced stages of pregnancy represented by time points T3 and T4.

Therefore, the most frequent adjustment of the LMWH dose was recommended in T3 and T4.

AA in EXTEM after the postpartum period was steeper than in controls (*p* = 0.0007), indicating that hemostasis is not fully normalized even after 6–8 weeks after delivery. Therefore, hematologists should evaluate the termination of anticoagulant thromboprophylaxis on the individual basis taking into account not only the clinical state of the patient and the laboratory results of the standard coagulation tests but also consider the use of ROTEM as the useful, quick, and complex method for the assessment of the whole coagulation process available at the patient´s bedside with minimal delay [27].

We strongly hope that our results could contribute to the determination of the reference ranges for the high-risk population of pregnant women that have not been established for the time period from the beginning of pregnancy up to 6–8 weeks after the delivery up to now.

We are aware of the several limitations of our study—such inaccuracy may be caused by the use of non-pregnant women as the control group, limited amount of included patients, especially in T1 due to the later visit at the hematologist or because of personal and health reasons. Moreover, cut-off values for the ROTEM^®^ parameters used for the evaluation of our results were determined using non-pregnant population and should be modified according to the results of the large prospective longitudinal studies performed in high-risk pregnant women. Anyway, we keep on including the patients and hope that we will contribute to better knowledge in this field of study also with a broader amount of data in the future.

## Figures and Tables

**Table 1:** General definitions and characteristics of the measured parameters and their reference ranges for particular tests used in the study ([10,11,12,13,14,15,16,17,18], r ex-tem^®^, Pentapharm GmbH, in-tem^®^, Pentapharm GmbH, hep-TEM^®^, Pentapharm GmbH).

	CT (s)	CFT (s)	AA	MCF (mm)
General characteristic	the time from the beginning of detection up to the initiation of clotting (clot firmness of 2 mm)	the time measured from the initiation of clotting until a clot firmness of 20 mm is achieved	the tangent to the graphic trace at the level of an amplitude of 2 mm	maximal amplitude of the graphical trace of clot firmness
EXTEM contains a stabilized preparation of human recombinant tissue factor acting as an activator and optimized amount of phospholipids. It is used for the assessment of the extrinsic pathway of coagulation (r ex-tem^®^, Pentapharm GmbH), providing data similar to that of the prothrombin time (PT)	38–79	34–159	63–83	50–72
INTEM is sensitive to heparin and can detect heparin levels from 0.2 to approximately 1.0 IU/mL. Reagent containing phospholipid and ellagic acid as contact activators provides data similar to activated partial thromboplastin time (aPTT). Therefore, the assay is used for the evaluation of the hemostatic system via intrinsic activation.	100–240	30–110	70–83	50–72
HEPTEM uses optimized calcium ion concentration in a buffer. Coagulation is initiated via the intrinsic pathway and lyophilized heparinase in the reagent rapidly degrades heparin in vitro. Therefore, when used in the association with INTEM reagent and compared to the results of INTEM analysis, it enables the detection of hemostatic status in heparinized patients without the effect of heparin.	137–246	40–100	NA	52–72

Legend: AA—alpha angle, aPTT—activated partial thromboplastin time, CFT—clot formation time, CT—clotting time, MCF—maximum clot firmness, NA—not available, PT—prothrombin time.

**Table 2:** Characteristics of pregnant patients and controls included in the study.

Mean Age (Age Range) (Controls)	Mean Age (Age Range) (Patients)	
29.42 (18–45)	30.24 (19–40)	
Mean weight (kg) (controls)	Mean weight (kg) (patients)	
65.5	T1	65.17
	T4	75.29
**RBC (Median (IQR)) (×10^12^/L) (Controls)** **(Reference Range 3.8–5.2 × 10^12^/L)**	**RBC (Median (IQR)) (×10^12^/L) (Patients)** ** (Reference Range 3.8–5.2 × 10^12^/L)**	
4.42 (4.16, 4.57)	T1	4.20 (4.17,4.23)
	T2	4.03 (3.97,4.10)
	T3	3.84 (3.75,3.96)
	T4	3.99 (3.84,4.08)
	T5	4.44 (4.24,4.67)
**Hgb (Median (IQR)) (g/L) (Controls) ** **(Reference Range 120–155 g/L)**	**Hgb (Median (IQR)) (g/L) (Patients)** ** (Reference Range 120–155 g/L)**	
132 (128, 138)	T1	128 (127,131)
	T2	122 (116,125)
	T3	118 (113,122)
	T4	122 (117,127)
	T5	133 (128,138)
**PLT (Median (IQR)) (×10^9^/L) (Controls)** ** (Reference Range 140–400 × 10^9^/L)**	**PLT (median (IQR)) (×10^9^/L) (Patients)** ** (Reference range 140–400 × 10^9^/L)**	
249 (202, 290)	T1	244 (213,273)
	T2	210 (207,226)
	T3	217 (175,230)
	T4	198 (171,236)
	T5	228 (196,278)
**Fbg (Median (IQR)) (g/L) (Controls)** ** (Reference Range 1.8–4.2 g/L)**	**Fbg (Median (IQR)) (g/L) (Patients)** ** (Reference Range 1.8–4.2 g/L)**	
2.70 (2.28, 3.21)	T1	3.16 (3.07,3.20)
	T2	3.07 (3.01,3.32)
	T3	3.54 (3.30,4.43)
	T4	4.27 (3.20,4.78)
	T5	2.54 (2.31,2.92)
**Change of the Dose of LMWH (%)**	T1	10.17
	T2	18.65
	T3	35.59
	T4	30.51
	T5	5.08
**Type of Delivery**	**Vaginal**	
	60.98% of the patients	
	**Cesarean Section**	
	39.02% of the patients	

Legend: IQR—interquartile range, Fbg—fibrinogen, LMWH—low-molecular-weight heparin, PLT—platelets, RBC—red blood cells.

**Table 3:** Descriptive statistics of CT, CFT, AA, and MCF in EXTEM in high-risk patients during pregnancy, after the postpartum period, and in the control group. Data are presented in the form of medians and interquartile ranges.

Variable	Co	T1	T2	T3	T4	T5
CT	67 (6 67 (60,70)	60 (55,66)	63 (60,68)	62 (58,66)	67 (56,72)	62 (58,70)
CFT	83 (74,96)	76 (64,95)	74 (69,76)	61 (55,67) ***	63 (58,69) **	73 (60,84)
NA in CFT	0	0	0	0	0	2
AA	73.0 (71.0,75.0)	74.5 (71.2,77.0)	75.5 (74.8,77.2)	78.0 (77.0,79.0) ***	77.0 (76.5,79.0) ***	76.0 (74.0,78.0) ***
NA in AA	0	0	0	0	0	1
MCF	63.0 (61.0,65.0)	65.5 (62.8,67.8)	67.0 (63.0,69.5)	69.0 (66.0,72.0) ***	70.0 (66.5,72.0) ***	64.5 (63.0,67.5)

Legend: AA—alpha angle, CFT—clot formation time, Co—control group, CT—clotting time, MCF —maximum clot firmness, NA—not available (missing data), T1—time interval 1, T2—time interval 2, T3—time interval 3, T4—time interval 4, T5—time interval 5. Significant differences of the results between T1–T5 and controls are highlighted with asterisk sign according to the following key: * <0.05, ** 0.01 and *** <0.001. *p*-values are related to ANOVA test.

**Table 4:** Development of CT, CFT, AA, and MCF values in INTEM in high-risk patients during pregnancy, after the postpartum period, and in the control group. Data are presented in the form of medians and interquartile ranges.

Variable	Co	T1	T2	T3	T4	T5
CT	173 (158, 184)	186 (174, 220)	162 (157, 210)	163(144, 176)	157 (151, 171)	177 (163, 181)
CFT	73 (64, 83)	88 (73, 111) *	70 (64, 75)	66 (52, 72)	58 (54, 68) **	68 (63, 79)
AA	75.0 (74.0, 77.0)	72.0 (68.0, 75.2)	76.5 (75.0, 77.2)	77.0 (75.5, 79.0)	78.0 (77.0, 79.0) **	76.0 (74.0, 77.2)
MCF	60.0 (58.0, 63.0)	61.5 (57.8, 63.2)	64.5 (60.5, 66.2)	67.0 (64.0, 70.0) ***	68.0 (65.0, 71.0) ***	61.5 (59.8, 64.5)

Legend: AA—alpha angle, CFT—clot formation time, Co—control group, CT—clotting time, MCF—maximum clot firmness, T1—time interval 1, T2—time interval 2, T3—time interval 3, T4—time interval 4, T5—time interval 5. Significant differences of the results between T1–T5 and controls are highlighted with asterisk sign according to the following key: * <0.05, ** <0.01 and *** <0.001. *p*-values are related to ANOVA test.

**Table 5:** Dynamics of CT, CFT, AA, and MCF values in HEPTEM in high-risk patients during pregnancy, after the postpartum period and in the control group. Data are presented in the form of medians and interquartile ranges.

	Co	T1	T2	T3	T4	T5
CT	169 169 (156, 192)	175 (166, 185)	177 (165, 201)	160 (150, 174)	165 (158, 175)	178 (165, 195)
CFT	72 (69, 86)	77 (70, 82)	76 (71, 80)	64 (53, 72)	62 (55, 8)	80 (66, 3)
AA	75.0 (73.0, 76.0)	75.0 (74.0, 76.0)	74.5 (74.0, 76.2)	77.0 (75.5, 79.0) *	77.0 (74.2, 79.0)	74.0 (72.0, 76.5)
NA in AA	0	0	0	0	0	1
MCF	59.0 (57.0, 62.0)	60.0 (58.8, 61.2)	60.0 (58.8, 62.8)	65.0 (63.0, 68.5) **	67.0 (64.2, 70.5) ***	61.0 (56.0, 62.5)

Legend: AA—alpha angle, CFT—clot formation time, CT—clotting time, MCF—maximum clot firmness, NA—not available (missing data), T1—time interval 1, T—time interval 2, T3—time interval 3, T4—time interval 4, T5—time interval 5. Significant differences of the results between T1–T5 and controls are highlighted with asterisk sign according to the following key: * <0.05, ** <0.01 and *** <0.001. *p*-values are related to ANOVA test.
